# Early transcriptional responses of bronchial epithelial cells to whole cigarette smoke mirror those of *in-vivo* exposed human bronchial mucosa

**DOI:** 10.1186/s12931-022-02150-2

**Published:** 2022-09-02

**Authors:** Anne M. van der Does, Rashad M. Mahbub, Dennis K. Ninaber, Senani N. H. Rathnayake, Wim Timens, Maarten van den Berge, Hananeh Aliee, Fabian J. Theis, Martijn C. Nawijn, Pieter S. Hiemstra, Alen Faiz

**Affiliations:** 1 Department of Pulmonology, Leiden University Medical Center, Leiden, The Netherlands; 2 Respiratory Bioinformatics and Molecular Biology (RBMB), School of Life Sciences, University of Technology Sydney, Sydney, Australia; 3 Department of Pathology and Medical Biology Diseases, University of Groningen, University Medical Center Groningen, Groningen, The Netherlands; 4 University of Groningen, University Medical Center Groningen, Groningen Research Institute for Asthma and COPD, Groningen, The Netherlands; 5 Department of Pulmonary Diseases, University of Groningen, University Medical Center Groningen, Groningen, The Netherlands; 6 Institute of Computational Biology, Helmholtz Centre, Munich, Germany

## Abstract

**Background:**

Despite the well-known detrimental effects of cigarette smoke (CS), little is known about the complex gene expression dynamics in the early stages after exposure. This study aims to investigate early transcriptomic responses following CS exposure of airway epithelial cells in culture and compare these to those found in human CS exposure studies.

**Methods:**

Primary bronchial epithelial cells (PBEC) were differentiated at the air–liquid interface (ALI) and exposed to whole CS. Bulk RNA-sequencing was performed at 1 h, 4 h, and 24 h hereafter, followed by differential gene expression analysis. Results were additionally compared to data retrieved from human CS studies.

**Results:**

ALI-PBEC gene expression in response to CS was most significantly changed at 4 h after exposure. Early transcriptomic changes (1 h, 4 h post CS exposure) were related to oxidative stress, xenobiotic metabolism, higher expression of immediate early genes and pro-inflammatory pathways (i.e., Nrf2, AP-1, AhR). At 24 h, ferroptosis-associated genes were significantly increased, whereas *PRKN*, involved in removing dysfunctional mitochondria, was downregulated. Importantly, the transcriptome dynamics of the current study mirrored *in-vivo* human studies of acute CS exposure, chronic smokers, and inversely mirrored smoking cessation.

**Conclusion:**

These findings show that early after CS exposure xenobiotic metabolism and pro-inflammatory pathways were activated, followed by activation of the ferroptosis-related cell death pathway. Moreover, significant overlap between these transcriptomic responses in the *in-vitro* model and human *in-vivo* studies was found, with an early response of ciliated cells. These results provide validation for the use of ALI-PBEC cultures to study the human lung epithelial response to inhaled toxicants.

**Supplementary Information:**

The online version contains supplementary material available at 10.1186/s12931-022-02150-2.

## Introduction

Cigarette smoking remains the principal preventable cause of death (eight million deaths/year) associated with multiple diseases, including chronic obstructive pulmonary disease (COPD), lung cancer, and cardiovascular diseases [1–5]. The respiratory epithelium is the first line of defence against inhaled pathogens and toxicants, including cigarette smoke (CS). Billatos and colleagues demonstrated that individuals without recent CS exposure developed an altered epithelial gene expression profile 24 h after smoking three cigarettes [6], highlighting the rapid effects of CS on the airway epithelium. Moreover, numerous studies have shown that the gene expression profile of the airway epithelium is markedly different in active smokers [7–11]. Smoking cessation studies have revealed that altered expression of most smoking-related genes is transient and normalises over time to non-smoker level. There is, however, a subset of genes that remains persistently altered, possibly related in part to lung tissue remodelling [7, 12].

In order to understand the physiological and pathological processes resulting from CS, it is pivotal to unravel the complex dynamics of the transcriptional changes already in the early phase after exposure, as well as to better understand how they relate to the development of long-term effects, such as impaired mucociliary clearance and tissue remodelling. Especially the characterization of immediate early genes (IEGs) remains understudied and could give interesting insights into the rapid cellular response possibly dictating the long-term effects and immune responses. IEGs are a collection of genes that are induced within minutes to hours by various triggers as these do not require de novo protein synthesis, and many are transcription factors or other DNA-binding proteins involved in transcription of genes involved in later responses [13, 14]. Human studies pose limitations into obtaining mechanistic insight in this very early phase. On the other hand, the translation of results from animal studies to humans is often problematic [15]. *In-vitro* models can therefore be more suitable, but their relevance is very dependent on the set-up. CS consists of more than 7000 chemical components, of which 158 are considered as toxicants [16, 17]. These toxicants are distributed between the particulate and gaseous phases. However, *in-vitro* research on CS traditionally focuses on cell cultures which are exposed to cigarette smoke components dissolved in a medium under submerged conditions [18]. Whole CS exposure systems can capture the full interaction between the particulate and gaseous phase of CS but require a cell culture that is exposed to air. Therefore, various studies have now successfully used whole CS exposure of primary human airway epithelial cells cultured at the physiological relevant air–liquid interface (ALI) [19, 20]. Furthermore, these ALI cultures allow epithelial differentiation to a mucociliary layer that closely mimics the cellular makeup and function of the airway epithelium in situ in contrast to conventional undifferentiated, submerged culture [21]. A direct comparison of results obtained using such systems to epithelial changes in smokers is an important step in the validation of cell culture models to study the epithelial response to inhaled toxicants such as cigarette smoke, and would, furthermore, help to promote transition of animal experimentation to *in-vitro* models.

The aim of the present study is to first perform an in-depth transcriptomic analysis of the early and late response of human primary differentiated airway epithelial cell cultures to whole cigarette smoke. Additionally, we aimed to compare these results to datasets obtained from human CS studies to validate the use of these models.

## Methods

### Study population and sample selection

Cells were isolated from macroscopically normal lung tissue obtained from patients undergoing resection surgery for lung cancer at the Leiden University Medical Center, the Netherlands. Patients (N = 8) from which this lung tissue was derived were enrolled in the biobank via a no-objection system for coded anonymous further use of such tissue (www.federa.org). However, since 29-11-2020, patients are enrolled in the biobank using active informed consent in accordance with local regulations from the LUMC biobank with approval by the institutional medical ethical committee (B20.042/Ab/ab and B20.042/Kb/kb).

Patients were diagnosed with lung cancer and had varying smoking status, including smokers, ex-smokers, and non-smokers. No participants were clinically diagnosed with COPD or were receiving oral or inhaled steroids at the time of tissue collection. A summary of the participants’ characteristics is provided in Table 1.

**Table 1:** Donor characteristics

Number of participants	8
Male/Female	7/1
Age (years) mean [SD]	66.6 [6.2]
BMI mean [SD]	25.5 [3.7]
Smoking Status (non-/ex-/smokers)	1/3/4
FEV_1_/VC mean [SD]	68.9 [11.7]

*SD* standard deviation

### ALI-PBEC culture preparation and RNA-sequencing

**Fig. 1:**
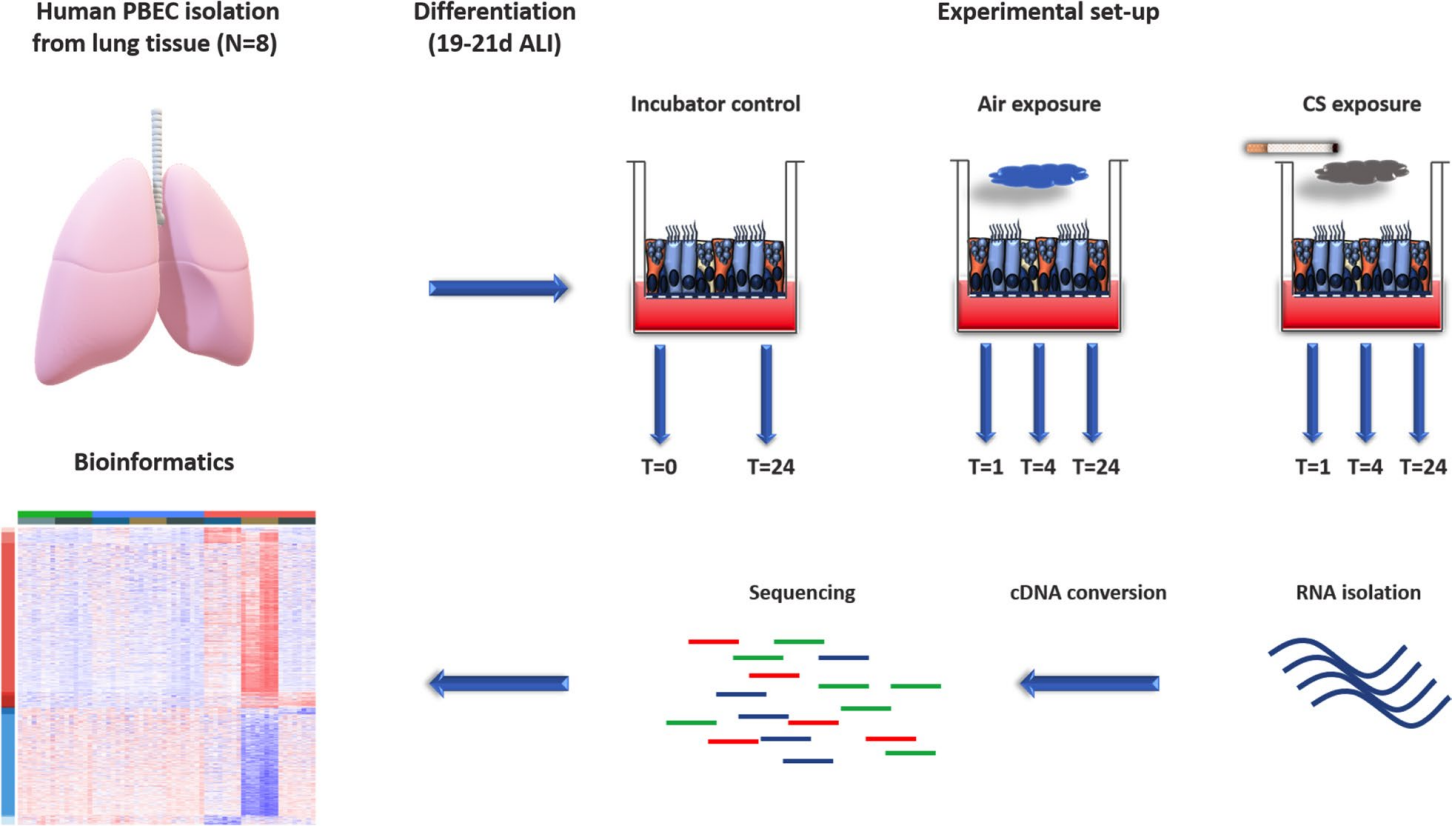
Experimental design of ALI-PBEC study. Primary bronchial epithelial cell cultures were derived from resected lung tissue (n = 8) and differentiated at the air–liquid interface (ALI). Differentiated cultures were exposed once to whole cigarette smoke (CS) or Air and samples were collected at 1 h, 4 h and 24 h after exposure for RNA-sequencing and ELISA. Bioinformatics analysis was performed on the RNA-seq dataset. Incubator controls were kept untouched in the cell culture incubator during the whole procedure. DGE: differential gene expression, GSVA: gene set variation analysis, PBEC: primary bronchial epithelial cell, ALI: Air–liquid interface

PBEC were differentiated in ALI and were exposed to whole cigarette smoke (CS), freshly generated from one cigarette, or air (Air) as control for 4–5 min. Cells were harvested for RNA-sequencing at 1 h, 4 h, and 24 h after exposure. The detailed description of the processing and differentiation of the ALI-PBEC culture, the CS/Air set-up, and RNA-sequencing is described in the online supplement (Additional file 1). A method flowchart is provided in Fig. 1.

### Differential gene expression

Differential gene expression analysis was performed between CS and Air in a paired analysis using the edgeR package (version 3.30.3) in R (version 4.0.2). To correct for multiple testing, the false discovery rate (FDR) was set to 5% using the Benjamini–Hochberg procedure. Fold change (FC) above 2 and below -2 was considered significantly up- and downregulated, respectively. The associated pathways were identified in gprofiler (https://biit.cs.ut.ee/gprofiler/gost). Gene set variation analysis (GSVA) was performed on the differentially expressed genes (DEGs) and identified pathways using the GSVA package (version 1.36.2) in R (version 4.0.2). The differential gene expression and pathway analysis is described in detail in the online supplement (Additional file 1).

### Cell-type deconvolution

Cell-type deconvolution analysis was performed to associate gene signatures with epithelial cell types, and is described in the online supplement (Additional file 1) [22].

### Comparative analysis with smoke-related in-vivo and in-vitro studies

Gene expression analysed in a number of previously published *in-vivo* human CS-related studies were compared with the current *in-vitro* study. The selected studies were: (i) an acute cigarette smoke exposure study (“Impact of acute exposure to cigarette smoke on airway gene expression”; ClinicalTrials.gov, Identifier: NCT00850863) [6]. In this study, airway epithelial brushings were collected from 63 individuals without recent smoke exposure (two days or longer) 24 h after smoking three cigarettes. Baseline bronchial brushings were collected six weeks or more post-CS exposure. DGE was performed between the baseline and post-CS exposure (FC >|1.5|, FDR < 0.05). The threshold was lower in the study as there were no significant genes at FC >|2|. The second data set was used from (ii) a chronic cigarette smoke exposure study (“Nasal epithelium as a proxy for bronchial epithelium for smoking-induced gene expression and expression Quantitative Trait Loci”; ClinicalTrials.gov, Identifier: NCT00848406) [23]. In this study bronchial biopsies were collected from 42 healthy chronic smokers and 35 never smokers. DGE was performed between chronic smokers and never smokers (FC >|2|, FDR < 0.05). Lastly, (iii) data from a smoke cessation study (“Effect of 1-year smoking cessation on airway inflammation in COPD and asymptomatic smokers”) was used [24, 25]. In this study bronchial biopsies were collected from 16 cigarette smokers before and after one year of smoking cessation. DGE analysis was performed between pre- and post-cessation (FC >|2|, FDR < 0.05). Expression of these differentially expressed genes from each of the mentioned *in*-*vivo* human studies was analysed by GSVA package (version 1.36.2) in R (version 4.0.2). Furthermore, we also compared our DEGs with (iv) publicly available single-cell sequencing of bronchial brushings collected from healthy never smokers (n = 6) and current smokers (n = 6) (“Characterizing smoking-induced transcriptional heterogeneity in the human bronchial epithelium at single-cell resolution”, GSE131391) [26]. Signature expression of the DEGs in the current *in-vitro* study was assessed in the single-cell *in-vivo* study. The analysis was performed using Seurat package (version 4.0.2) in R (version 4.0.5). Gene expression measurements were normalised using the global-scaling normalisation method, ‘LogNormalize’, and linear transformation was applied using the highly variable genes identified by the variance-stabilising method. The dimensional reduction was performed using principal component analysis, and the cells were clustered at 0.2 resolution using the Louvain algorithm. To visualise the dataset, the nonlinear dimensionality reduction technique UMAP was selected. Cell types were identified using the canonical marker genes, which were also used in the respective in-vivo study.

Additionally, we also compared our study with two e-cigarette related studies. One is (v) an *in-vitro* study (“Molecular impact of electronic cigarette aerosol exposure in human bronchial epithelium”, GEO accession: GSE82137), where we performed GSVA on the DEGs between e-cigarette (with or without nicotine) and air, using the gene expression dataset of the current study [27]. In this study, differentiated ALI-PBECs, collected from the lungs of a single healthy male donor, were exposed to whole CS, e-cigarette aerosol (with and without nicotine) or air. DGE was performed between CS/e-cigarette and air (FC >|2|, FDR < 0.05). The other study was (vi) an *in-vivo* e-cigarette study where bronchial epithelial cells were collected from former cigarette smokers (n = 21), e-cigarette users (n = 15), and current cigarette smokers (n = 9) (“Gene Expression Alterations in Bronchial Epithelium of Electronic Cigarette Users”, GEO accession: GSE112073) [28]. The study was an observational study. However, in this study no differentially expressed genes were identified between e-cigarette smokers and former smokers (FC >|2|, FDR < 0.05). Therefore, we used DEGs identified in the current study at 24 h between CS and Air and performed GSVA using the gene expression dataset of the *in-vivo* e-cigarette smoke study.

### Statistical analysis

Analyses of the significance of differences between CS and Air gene signatures using GSVA were performed using two-way ANOVA with Sidak’s multiple comparisons test in Prism 8 (version 8.4.3).

## Results

### Transcriptional profiling of the response of differentiated ALI-PBEC to whole CS

**Fig. 2:**
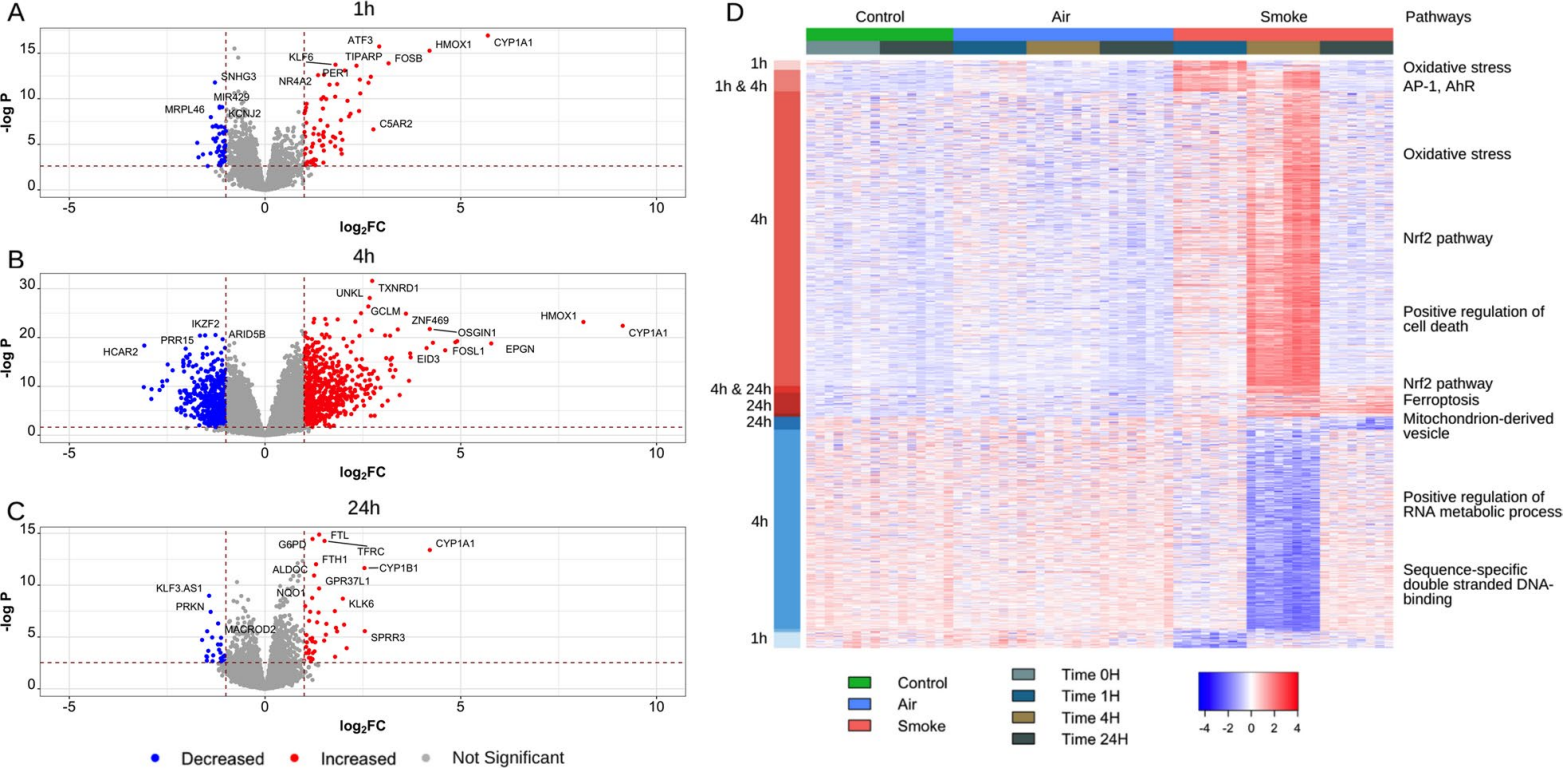
Differential expression of genes after whole cigarette smoke exposure of primary bronchial epithelial cells differentiated at the air–liquid interface. Gene expression was assessed by RNA-seq in well-differentiated primary bronchial epithelial cells (PBEC; n = 8 individual donors) grown at the air–liquid interface (ALI) at several time-points after whole cigarette smoke exposure. The volcano plots show DEGs (adj. p-value < 0.05, FC >|2|) upon smoke exposure at **A** 1 h (112 genes), **B** 4 h (1048 genes), and **C** 24 h (57 genes). The red dots indicate the upregulated genes, and the blue dots indicate the downregulated genes. **D** Heatmap of DEGs upon cigarette smoke exposure. Red and blue in the row sidebar indicate upregulated and downregulated genes, respectively

Mucociliary-differentiated ALI-PBEC cultures were generated from 8 different donors and exposed to whole CS or Air. At several time-points hereafter (1 h, 4 h and 24 h), RNA-sequencing was performed to measure total gene expression (Fig. 1). At 1 h after exposure, 72 genes were significantly increased in expression when comparing CS to Air, and 40 genes were significantly reduced in expression (FDR < 0.05, FC >|2|). Within the set of 72 increased genes, a variety of IEGs were identified including *NRFA1* and *2*, *FOS*, *FOSB, JUN, ATF3*, and *EGR-1*. In addition, a number of xenobiotic metabolism-associated genes were identified, including the well-known *CYP1A1* and *CYP1B1* [29, 30]. The highest number of DEGs across all exposures was found after 4 h when 633 genes were significantly increased in expression comparing CS to Air, and 415 genes were significantly reduced in expression (FDR < 0.05, FC >|2|). At 4 h, genes associated with the Nrf2 pathway such as *TXNRD1, GSR, HMOX1,* and the inflammatory mediator *CXCL8* were significantly increased [31, 32]. At 24 h, we found 68 DEGs, comparing CS to Air (42 increased, and 26 decreased (FDR < 0.05, FC >|2|)). Ferroptosis-related genes, such as, *FTL, TFRC, FTH1,* were actively transcribed, and *PRKN*, which plays a central role in removing dysfunctional mitochondria, was significantly downregulated in response to CS [33, 34]. The complete dataset is summarised in Fig. 2. The top DEGs for each time points are provided in Table 2 and Additional file 2: Table S1 respectively. ALI-PBEC cultures were exposed in two independent experiments (n = 4 donors each) with minor variation in overall smoke exposure (2.1 mg and 3.9 mg). A total of 49 and 92 genes were found to be different between the two batches at 1 h and 4 h, respectively, while no genes differed after 24 h of exposure (Additional file 1: Figure S1).

**Table 2:** Top 15 DEGs at 1 h, 4 h, and 24 h after whole cigarette smoke exposure

Gene symbol	logFC	logCPM	Adj. P-value	FDR
*1 h*				
CYP1A1	5.69	8.73	1.23E−17	1.76E−13
ATF3	2.92	6.41	2.91E−16	1.39E−12
HMOX1	4.20	9.42	4.78E−16	1.71E−12
FOSB	3.16	4.71	8.43E−15	2.01E−11
KLF6	1.80	6.62	1.52E−14	3.10E−11
TIPARP	2.34	7.06	1.89E−14	3.38E−11
ZNF331	2.04	4.57	7.22E−14	1.15E−10
PER1	1.51	6.00	1.92E−13	2.74E−10
NR4A2	1.36	6.20	2.73E−13	3.55E−10
CYP1B1	2.70	8.93	3.27E−13	3.90E−10
SNHG3	-1.27	3.93	7.38E−13	8.12E−10
NR4A1	2.43	6.25	8.27E−13	8.45E−10
CBARP	2.64	2.00	1.41E−12	1.34E−09
OVOL1	1.65	3.56	1.70E−12	1.52E−09
RHOB	1.84	6.31	2.92E−12	2.46E−09
*4 h*				
TXNRD1	2.76	9.97	1.38E−32	1.97E−28
UNKL	2.70	5.80	2.72E−29	1.94E−25
GCLM	2.66	7.79	1.23E−26	5.87E−23
SQSTM1	2.47	10.51	1.80E−26	6.45E−23
STX3	1.28	6.34	4.49E−26	1.28E−22
ZNF469	3.62	5.31	1.38E−25	3.30E−22
SYNJ2	1.88	6.97	2.31E−25	4.71E−22
GSR	1.26	7.67	9.42E−25	1.68E−21
SLC12A7	1.56	7.84	2.02E−24	3.20E−21
HMOX1	8.16	9.42	5.57E−24	7.96E−21
GCLC	2.33	8.75	6.23E−24	8.10E−21
CYP1A1	9.16	8.73	3.97E−23	4.38E−20
AKR1C3	1.60	7.27	3.98E−23	4.38E−20
CYP4F3	2.75	6.02	1.15E−22	1.13E−19
OSGIN1	4.23	6.66	1.19E−22	1.13E−19
*24 h*				
FTL	1.38	9.38	9.49E−16	9.61E−12
G6PD	1.21	7.97	1.34E−15	9.61E−12
TFRC	1.52	7.39	2.38E−15	1.13E−11
CYP1A1	4.21	8.73	4.23E−14	1.21E−10
FTH1	1.30	10.70	9.39E−13	1.92E−09
CYP1B1	2.54	8.93	1.92E−12	3.04E−09
ALDOC	1.25	3.90	6.35E−12	7.57E−09
GPR37L1	1.38	2.82	2.44E−10	1.74E−07
KLF3.AS1	− 1.43	3.76	1.22E−09	7.26E−07
KLK6	1.99	1.88	1.76E−09	9.00E−07
NQO1	1.20	8.47	2.87E−09	1.37E−06
ALDH3A1	1.03	10.37	1.29E−08	4.86E−06
SERPINB4	1.78	5.46	2.60E−08	9.05E−06
PRKN	− 1.39	1.04	2.98E−08	1.01E−05
AKR1B10	1.15	6.46	4.68E−08	1.49E−05

*logFC* log fold change, *logCPM* log counts per million, *adj*. adjusted, *FDR* false discovery rate

**Fig. 3:**
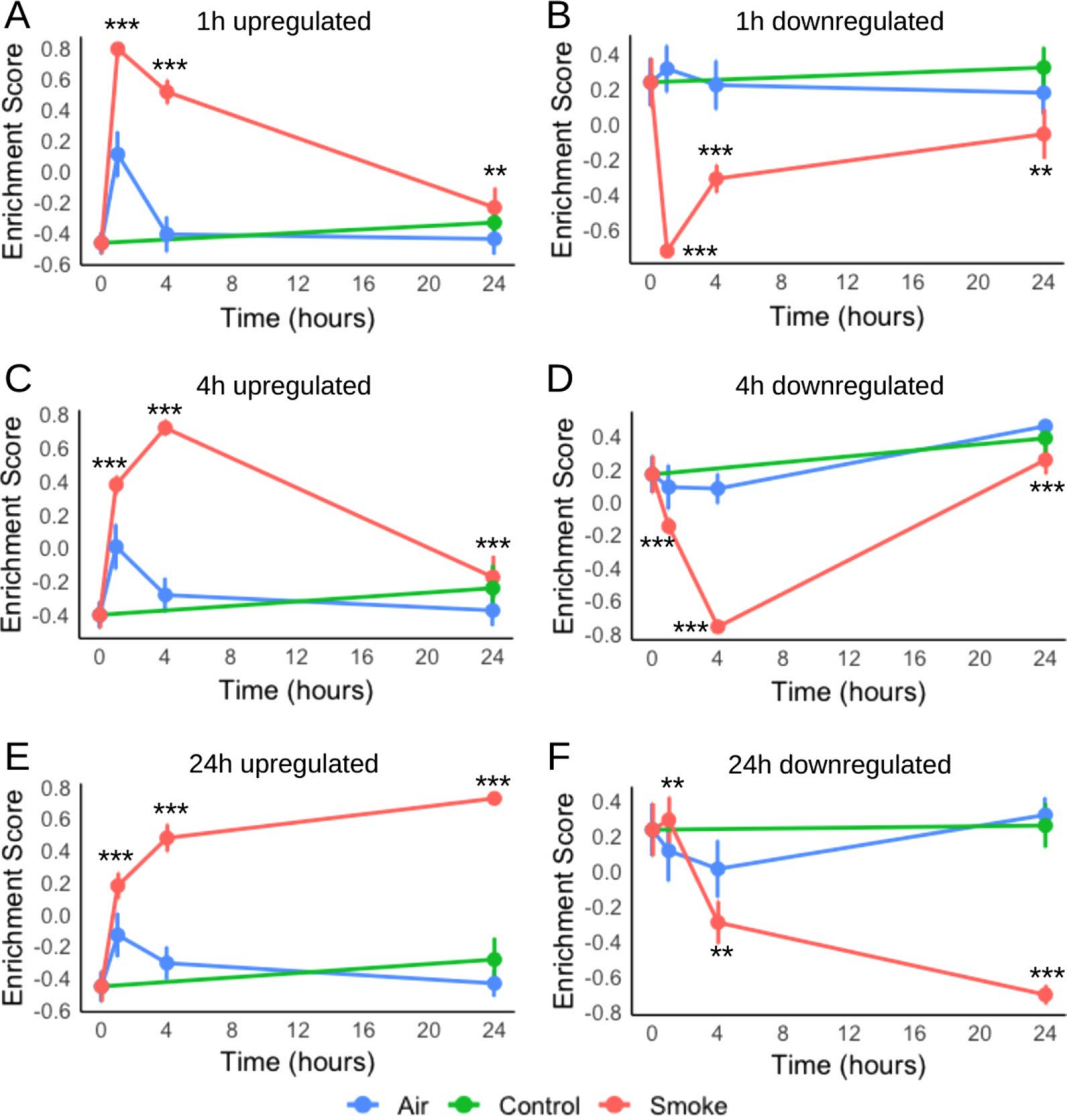
Time-point signatures of DEGs after whole cigarette smoke exposure of primary bronchial epithelial cells differentiated at the air–liquid interface. Gene set variation analysis (GSVA) was performed on DEGs after **A**, **B** 1 h, **C**, **D** 4 h, and **E**, **F** 24 h of exposure (n = 8 donors). The error bar indicates the standard deviation. Gene sets which were significantly different after cigarette smoke exposure compared to Air (p < 0.05) are shown by the * symbol. *p < 0.01, ***p < 0.001

To determine whether the genes that were significantly increased in expression at earlier time points remained differentially expressed at 24 h post-exposure, we conducted gene set variation analysis (GSVA). GSVA estimates the relative enrichment score for a gene-set per sample, instead of a single gene value for each sample. This facilitates the interpretation of large gene expression dataset. The results showed that early response genes turned on in the first hour were reduced by 4 h and completely turned off by 24 h (p < 0.05, Fig. 3A, B), whereas long-term responsive genes that remained differentially expressed by 24 h were already switched on within the first hour after exposure (Fig. 3C–F).

### Pathways associated with DEGs upon CS exposure

**Fig. 4:**
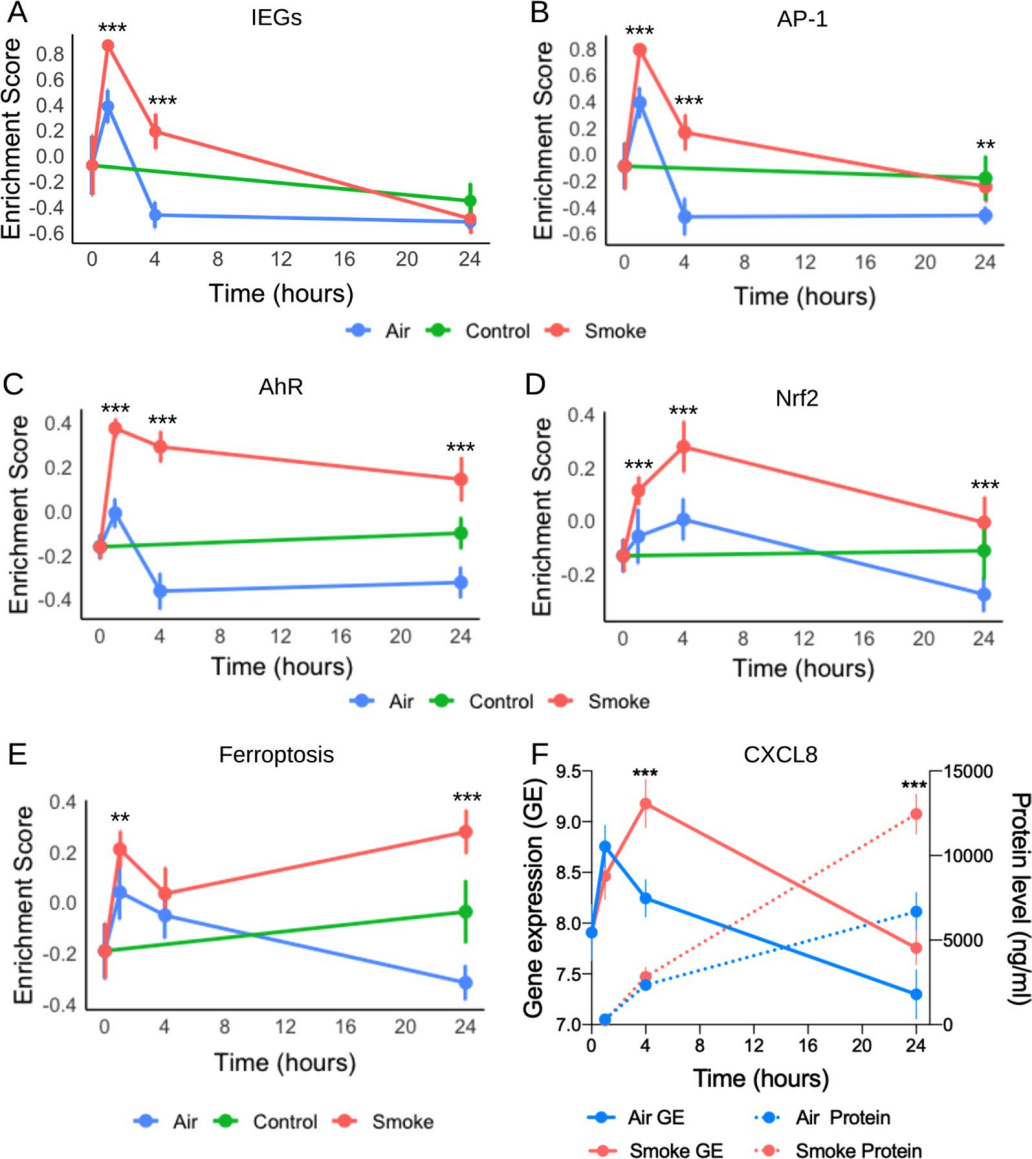
Pathway and protein level analysis of DEGs after whole cigarette smoke exposure of primary bronchial epithelial cells differentiated at the air–liquid interface. GSVA analysis of **A** immediate early genes (IEGs), and genes associated with **B** activator protein-1 (AP-1), **C** aryl hydrocarbon receptor (AhR), **D** Nrf2, **E** ferroptosis pathways. **F** Kinetics of *CXCL8* gene expression and CXCL8 protein level upon cigarette smoke exposure. The error bar indicates the standard deviation (n = 8). Gene sets which were significantly different after cigarette smoke exposure compared to Air (p < 0.05) are shown by the * symbol. **p < 0.01, ***p < 0.001

To investigate pathways associated with the observed changes in gene expression after CS exposure, we used gprofiler. This unbiased analysis revealed that at earlier time points (1 h and 4 h after exposure), pathway associated with oxidative stress, and xenobiotic metabolism were significantly upregulated (p < 0.05) (Table 3). Moreover, we selected a list of eight genes (*FOS, FOSB, JUN, NR4A1, NR4A2, MCL1, ATF3, EGR1*) which have been identified as immediate early genes (IEGs) across several publications [13, 35]. All of these genes except *MCL1* had their maximal expression at the 1 h time point with a reduction by the 4 h time point (Additional file 1: Figure S2). Furthermore, activator protein-1 (AP-1) and aryl hydrocarbon receptor (AhR)-related pathways were also significantly upregulated at 1 h after CS exposure (p < 0.05). GSVA on IEGs, AhR- and AP-1-pathway-related genes showed that expression was maximal at 1 h after CS and remained significantly enriched at all following time points compared to Air (p < 0.05) (Fig. 4A−C). At 4 h after CS exposure, nuclear factor erythroid 2-related factor 2 (Nrf2) signalling pathway was activated (p < 0.05, Fig. 4D). At 24 h after CS exposure, ferroptosis (a necrotic form of cell death) pathway was significantly upregulated by CS (p < 0.05) (Fig. 4E). A list of top associated pathways is provided in the supplementary material (Additional file 2: Table S2).

**Table 3:** Top pathways associated with the DEGs upon cigarette smoke exposure

Pathway description	Term ID	Database source	Adj. p-value
*1 h upregulated*			
DNA-binding transcription activator activity, RNA polymerase II-specific	GO:0001228	Gene ontology	2.42E−09
Positive regulation of macromolecule metabolic process	GO:0010604	Gene ontology	7.73E−08
Positive regulation of nitrogen compound metabolic process	GO:0051173	Gene ontology	2.84E−07
Response to hydrogen peroxide	GO:0042542	Gene ontology	4.12E−06
Response to organic cyclic compound	GO:0014070	Gene ontology	6.88E−06
Positive regulation of apoptotic process	GO:0043065	Gene ontology	8.93E−06
*4 h upregulated*			
Response to arsenic-containing substance	GO:0046685	Gene ontology	1.96E−07
Positive regulation of cell death	GO:0010942	Gene ontology	3.92E−05
Unsaturated fatty acid metabolic process	GO:0033559	Gene ontology	5.97E−05
Oxidative Stress	WP408	WikiPathways	1.77E−07
NRF2 pathway	WP2884	WikiPathways	3.44E−06
Glutamate-cysteine ligase activity	GO:0004357	Gene ontology	2.04E−04
*24 h upregulated*			
Nuclear Receptors Meta-Pathway	WP2882	WikiPathways	7.85E−06
NRF2 pathway	WP2884	WikiPathways	1.86E−05
Aryl Hydrocarbon Receptor Pathway	WP2873	WikiPathways	5.17E−05
Ferroptosis	KEGG:04216	KEGG	3.31E−05
Iron ion binding	GO:0005506	Gene ontology	2.73E−05
Oxidoreductase activity	GO:0016491	Gene ontology	1.11E−04
*4 h downregulated*			
Sequence-specific double-stranded DNA binding	GO:1990,837	Gene ontology	5.99E−06
Positive regulation of RNA metabolic process	GO:0051254	Gene ontology	8.11E−05
Positive regulation of nucleic acid-templated transcription	GO:1903508	Gene ontology	1.08E−04
*24 h downregulated*			
F-box domain binding	GO:1990444	Gene ontology	3.30E−02
Mitochondrion-derived vesicle	GO:0099073	Gene ontology	3.06E−02

No pathway was found related to 1 h downregulated genes

To further investigate the observation that the most significant pathway associated with 4 h of CS exposure was the Nrf2 pathway, we identified genes associated with this pathway from the publicly available data of Nrf2-siRNA treated A549 epithelial cells (GSE113519). GSVA showed that these Nrf2-associated genes were significantly enriched (p < 0.001) at 1 h, 4 h, and 24 h after CS compared to Air (Fig. 4D). Nrf2 is a transcription factor that regulates gene expression following its binding to antioxidant responsive elements and activates anti-oxidant genes [36]. Hence, we matched the positions of the Nrf2-associated genes to determine the proximity (± 50,000 bp) to known binding sites (obtained from publicly available ChIP-seq dataset, GSE75812). GSVA of genes thus identified to be associated with Nrf2 also showed significant differences (p < 0.001) at all three-time points after CS compared to Air (Additional file 1: Figure S3).

A well-known pro-inflammatory gene that was significantly increased in expression after 4 h of CS exposure was *CXCL8* (FDR < 3 × 10^–4^, Fig. 4F), which returned to base line by 24 h. This increase in *CXCL8* expression is likely the result of the suggested activation of the AP-1 pathway [37, 38]. Protein levels of CXCL8/IL-8 significantly increased by 24 h after CS exposure compared to Air (p < 0.05). These findings suggest transiently increased *CXCL8* gene expression at 4 h after CS exposure, followed by translation resulting in significantly increased CXCL8 levels at 24 h after CS exposure compared to Air.

### Assessment of impact of CS exposure on cell-type proportions in ALI-PBEC by cell-type deconvolution

**Fig. 5:**
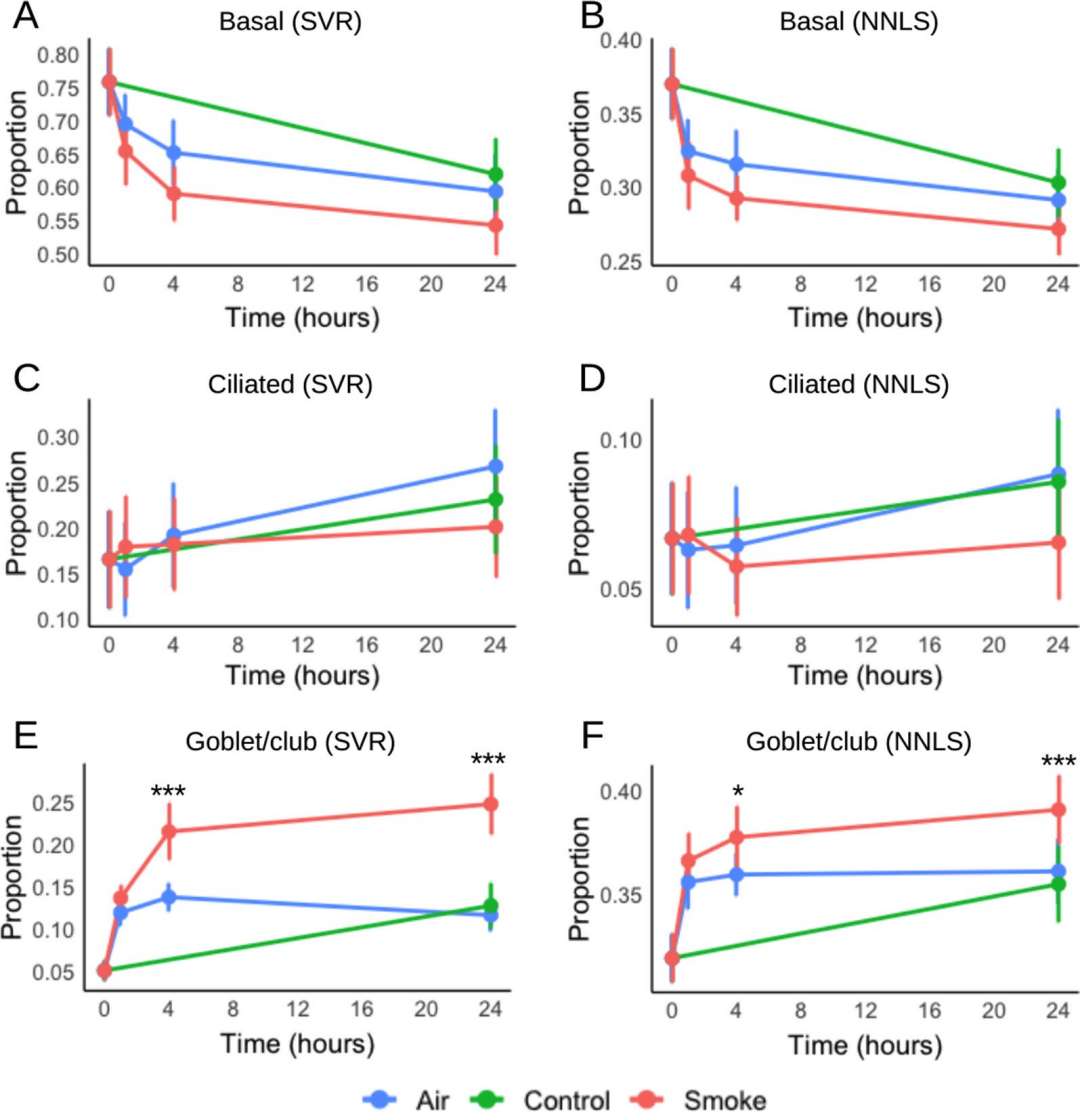
Cell-type deconvolution of primary bronchial epithelial cells differentiated at air–liquid interface during whole cigarette smoke exposure. Proportion of **A**, **B** basal, **C**, **D** ciliated, and **E**, **F** goblet/club cells based on gene-markers are shown here. The vertical error bar indicates the standard deviation (n = 8). Cell proportions which were significantly different after cigarette smoke exposure compared to Air (p < 0.05) are shown by the * symbol. *p < 0.05, ***p < 0.001. *SVR* support vector regression, *NNLS* non-negative least squares

Cell-type composition is an important factor in gene expression analysis, which is not often investigated in bulk RNA-Seq data [39]. Therefore, we conducted cell-type deconvolution using support vector regression (SVR) and non-negative least squares (NNLS) methods to assess the transcriptomic changes in cell-type proportions upon CS exposure. Initially we investigated the most abundant cell-types in our samples. Combined, basal, ciliated, and goblet/club cells had an average proportion greater than 75% in both SVR and NNLS methods (Additional file 1: Figure S4). The presence of different cell-types indicates that differentiation of our ALI-PBEC culture was successful, as no unexpected cell types were present. Next, we plotted a time-lapse graph to observe the change in proportions of these three cell-types. There was no significant difference in proportions of basal and ciliated cells upon CS exposure compared to Air (Fig. 5A–D). However, the proportion of goblet/club cell-expressing markers was significantly increased at 4 h and 24 h after CS exposure compared to Air in both SVR and NNLS methods (Fig. 5E, F). Although marker expression alone does not signify full differentiation, CS may have triggered the cellular machinery to start the differentiation process to goblet/club cells.

### Transcriptional changes in *in-vitro *ALI-PBEC model mirror those of *in-vivo* human CS studies

**Fig. 6:**
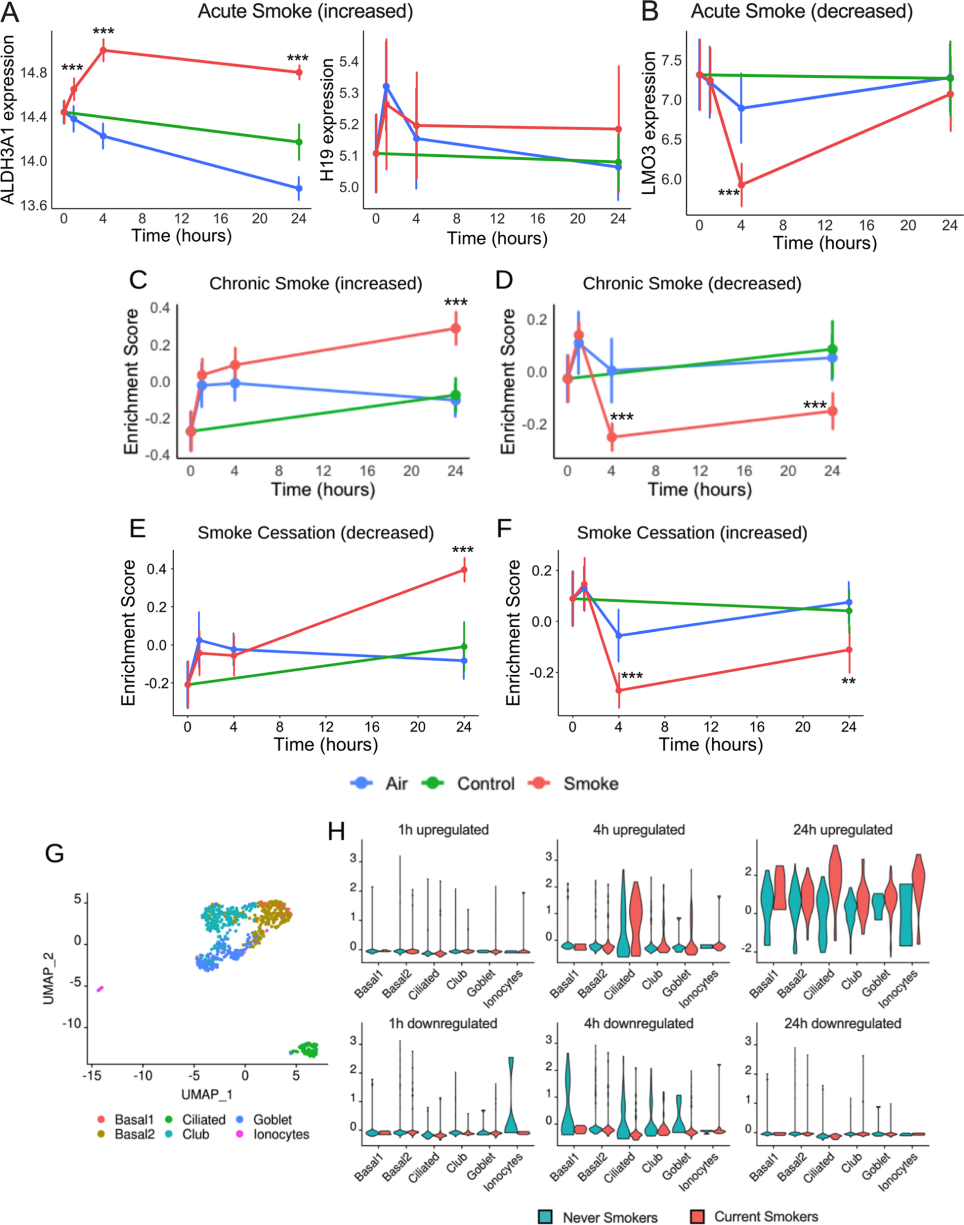
Comparison of cigarette smoke-related human *in-vivo* studies with the current *in-vitro* study. Expression of the genes that were differentially expressed in the **A**, **B** acute smoke exposure study is shown. Only two genes were upregulated and one gene was downregulated in the study. Gene set variation analysis (GSVA) of DEGs identified in *in-vivo* studies of **C**, **D** chronic smokers, and **E**, **F** smoking cessation is depicted here. The vertical bar indicates the standard deviation (n = 8). Gene sets which were significantly different after cigarette smoke exposure compared to Air (p < 0.05) are shown by the * symbol. *p < 0.05, ***p < 0.001. **G** UMAP of the identified cell-types of single-cell RNA-sequencing study of current smokers and never smokers, and **H** average expression of the DEGs identified in the *in-vivo* study

To validate the results of our ALI-PBEC model, we compared this with human *in-vivo* studies of CS exposure. First, we used the results of a previous acute smoke exposure study in which bronchial brushings were collected from 63 individuals, without recent CS exposure, 24 h after smoking three cigarettes [6]. GSVA was performed on differentially expressed genes identified in the *in-vivo* dataset. This analysis showed that genes identified in the *in-vivo* study that were increased or reduced in expression upon CS exposure were also significantly up- or downregulated 24 h after CS compared to Air (Fig. 6A, B).

Similarly, we compared our *in-vitro* ALI-PBEC study with the *in-vivo* chronic smoke study where the bronchial biopsies were collected from 42 asymptomatic chronic smokers and 35 never smokers, both with a normal lung function [23]. GSVA of the gene set obtained from this chronic smoker study showed that the significantly higher expressed genes in chronic smokers were also significantly increased at 24 h after CS in the current study when compared to Air (Fig. 6C). The lower expressed genes were significantly decreased at 4 h as well as 24 h after CS exposure in our ALI-PBEC study (Fig. 6D).

Furthermore, we compared our human *in-vitro* ALI-PBEC study with an *in-vivo* smoke cessation study where the bronchial biopsies were collected from 16 subjects before and after 1 year of smoking cessation [24, 25]. GSVA on the gene set showed that the downregulated genes in the cessation study were significantly upregulated at 24 h after CS exposure in the current ALI-PBEC study compared to Air (Fig. 6E). On the other hand, the upregulated genes in the cessation study were significantly downregulated at 4 h and 24 h after CS exposure (Fig. 6F).

Using these datasets, an overlap was found in gene expression related to oxidative stress, xenobiotic metabolism, AhR and Nrf2 pathways, as shown by a positive association between the *in-vitro* study and the former two *in-vivo* smoker studies, and a negative association with the *in-vivo* smoking cessation study (Additional file 2: Table S3).

Finally, to assess gene expression pattern at the single cell level, we compared the DEGs of the current *in-vitro* study with a publicly available human CS-related *in-vivo* single-cell RNA-sequencing study (Fig. 6G). Interestingly, we found that the upregulated genes at 4 h after CS exposure in the current *in-vitro* study were higher expressed in the ciliated cells in the single-cell *in-vivo* study in current smokers compared to never smokers. The upregulated genes at 24 h after exposure were higher in expression in all of the identified cell-types in the *in-vivo* study in current smokers compared to never smokers. We also observed a similar lower expression pattern in the *in-vivo* study for the downregulated genes of the current study.

### Similarities between effects of CS and e-cigarette smoke on epithelial cells *in-vivo* and *in-vitro*

**Fig. 7:**
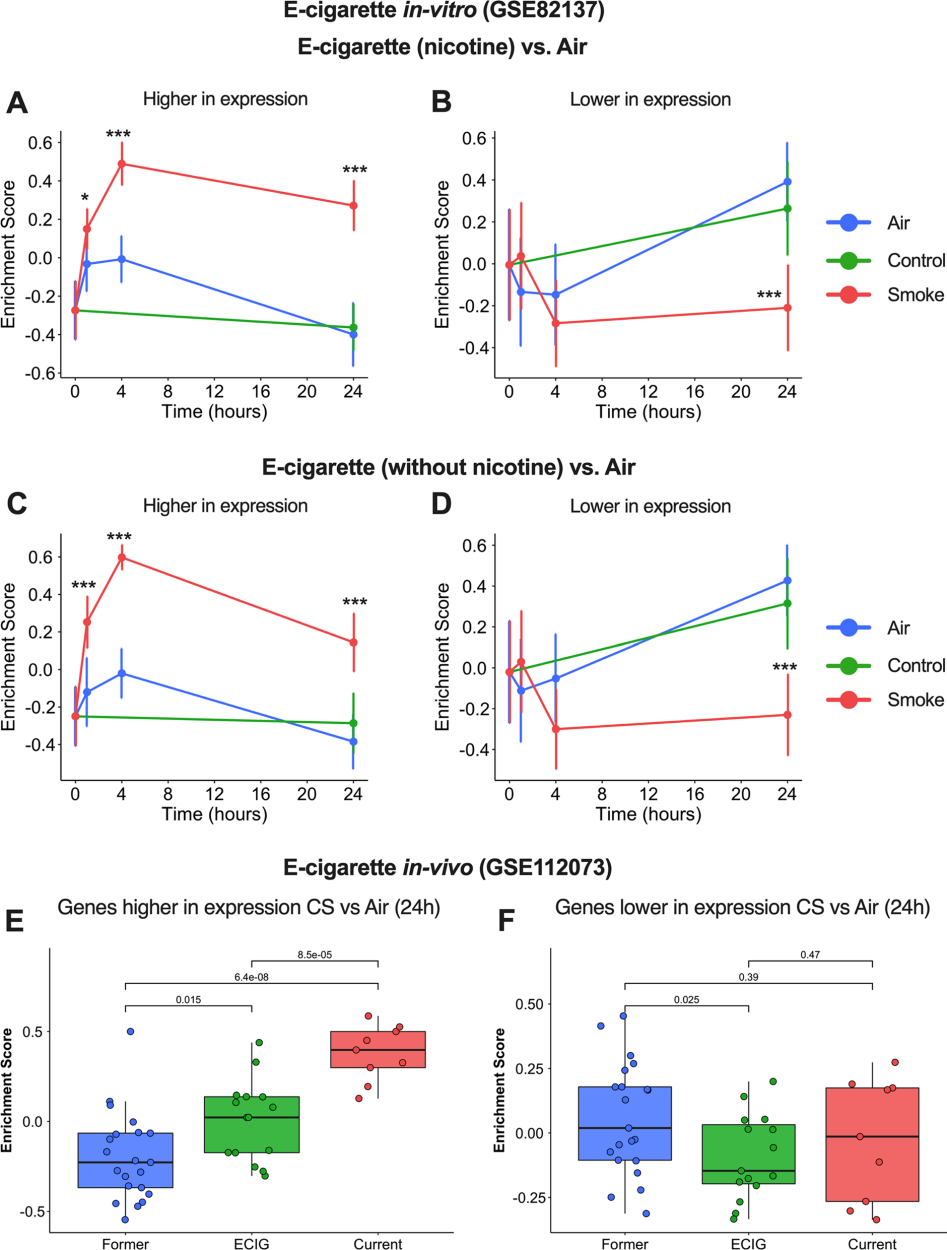
Transcriptomic analysis of e-cigarette smoke exposure or e-cigarette users in comparison with CS exposure. Gene set variation analysis (GSVA) of DEGs identified in *in-vitro* e-cigarette smoke study **A**, **B** with and **C**, **D** without nicotine. **E**, **F** GSVA of DEGs in the current study after CS exposure, plotted on the gene expression dataset of in-vivo e-cigarette smokers’ study. The vertical bar indicates the standard deviation. Gene sets which were significantly different after cigarette smoke exposure compared to Air (p < 0.05) are shown by the * symbol. *p < 0.05, ***p < 0.001

To compare the transcriptomic profile of CS exposure with that of e-cigarette, we analysed an e-cigarette *in-vitro* study in which PBECs, differentiated by ALI culture, were exposed to e-cigarette vapour or air, and gene expression changes were observed at 24 h after exposure. GSVA on DEGs, which displayed a higher expression after e-cigarette exposure, revealed a similar trend after CS exposure at all time points in the current CS study, regardless of the presence of nicotine in e-cigarette (Figs. 6C, 7A). In the case of DEGs, which were decreased in expression after e-cigarette exposure, the trend was significant at 24 h after CS exposure (Figs. 6D, 7B).

Subsequently, we compared the DEGs after CS exposure in the current study and investigated their expression in e-cigarette users. Hence, we performed GSVA using an *in-vivo* study where gene expression of bronchial epithelial cells, collected from former, current and e-cigarette smokers, were measured. GSVA on the DEGs at 24 h after CS exposure showed a similar trend in e-cigarette smokers compared to ex-smokers (Fig. 7E, F).

## Discussion

The current study shows that the transcriptional response of the airway epithelial cells to CS was initiated already after 1 h and was maximal at 4 h after exposure. This first, early wave involved immediate early response genes and was followed by genes associated with oxidative stress, xenobiotic metabolism and pro-inflammatory pathways, most of which remained active over time. At the 24 h time-point, expression of genes related to a necrotic form of cellular death, ferroptosis, was increased, which we speculate to have occurred in response to oxidative stress [33]. Additionally, changes in gene expression in response to CS were found to be associated with a higher proportion of goblet/club cells. The relevance of these findings is also supported by the significant overlap with a number of CS-related human *in-vivo* studies, providing important validation for the use of primary bronchial epithelial cell cultures differentiated at the air–liquid interface to support studies into kinetics of (whole) cigarette smoke-related events.

Exposure of ALI-PBEC cultures to CS activated several genes of the cytochrome P450 family, of which *CYP1A1* and *CYP1B1* genes were upregulated from the earliest time point till the latest at 24 h. This family of enzymes has been reported to be induced by polycyclic aromatic hydrocarbons (PAH), one of the carcinogens present in CS, and these P450 enzymes cause metabolic activation of PAH resulting in the formation of ROS [40]. Expression of various *CYP* genes in airway epithelial cells was shown to be dependent on their level of differentiation, underscoring the relevance for using well-differentiated cell cultures for these studies [40]. The peak of the increased expression for most genes by CS was at the earlier time points. For example, although Nrf2-associated genes were significantly upregulated throughout the measured 24 h, the expression of this gene set peaked at 4 h and decreased at 24 h after exposure. A similar pattern was seen for IEGs, AhR and AP-1. These data show the essence of capturing the early response to CS exposure as this can easily be missed when measuring gene expression only at 24 h after exposure. This could also explain why often changes in inflammation-related pathways remain undetected in studies focusing on those time points [6]. This is especially relevant in *in-vivo* studies, where it is often not possible to collect multiple samples so rapidly after the CS exposure. Alternatively, these cultures don’t represent the full tissue compartment in the airways, for example, in bronchial biopsy-derived studies where the multilayers of the tissue could have an influence over the pro-inflammatory response.

In the current study, *PRKN* was significantly downregulated at 24 h after CS exposure. This may be relevant in view of the central role of the PINK1-PRKN pathway in the removal of dysfunctional mitochondria through mitophagy [41]. Mitochondrial dysfunction is associated with a wide range of pathologies, including COPD [42]. Mitochondria release reactive oxygen species (ROS) as second messengers to maintain cellular homeostasis. However, dysfunctional mitochondria generate and accumulate an excessive amount of ROS leading to further impairment of mitochondrial function and increase in oxidative stress, cellular injury or cell death [43]. Moreover, decreased levels of the *PRKN*-encoded protein, parkin, has been reported in COPD lung tissue [44]. In a separate study, thickening of the airway wall (a characteristic feature of COPD) was shown in *PRKN*-deficient mice, as well as the accumulation of small-sized dysfunctional mitochondria in response to CS [34].

Importantly, our findings in differentiated ALI-PBEC cultures exposed to CS were in line with observations in smoking or ex-smoking human subjects. The DEGs identified after acute smoke exposure [6] and in chronic smokers [23] were found to be also differentially expressed in this study. In line with these findings, the gene signatures identified in the smoking cessation study were found to be reversed in the current ALI-PBEC study, i.e., downregulated genes in the smoking cessation study were significantly increased in our ALI-PBEC study and vice versa. This finding is expected as smoking cessation studies have shown that a subset of genes reverses back to the normal level after quitting smoking [7, 12]. Whereas here we investigated the acute CS response in a culture model comprised only of airway epithelial cells, the findings mirrored the pathways activated in response to CS of a much more complex cellular environment of the intact human bronchial mucosa sampled in the clinical bronchoscopy studies. Another study has provided evidence for a significant overlap in altered gene sets between an *in-vitro* set-up and human *in-vivo* exposure studies using a commercial airway cell culture system and cigarette smoke exposure combined with microarray analysis of the response of cells from a single donor [19]. However, our study demonstrates a significant overlap with findings from *in-vivo* exposures using multiple donors instead of a single donor, a different ALI culture protocol and different smoke set-up, as well as detailed RNA-seq on different time-points including the very early. These results show the robustness of the ALI culture set-up for in depth mechanistic investigations on effects of CS on the airway epithelium *in-vitro* and their translation in support of the transition into validated animal-free alternatives.

Moreover, in our study we have added the comparison with a single-cell RNA-sequencing *in-vivo* study revealing that ciliated cells, which are positioned at the apical side of the airway epithelium, mirror the expression of the DEGs that are found in our exposed cultures at 4 h, and in all other epithelial cell types by 24 h [26]. These are new insights that suggest ciliated cells to be early responders to external stimuli such as cigarette smoke and require further investigation. These results underscore the relevance of differentiated ALI-PBEC cultures and whole cigarette smoke exposure for understanding the complexity of the epithelial response to CS or any other toxicants and provide evidence for the robustness of these models.

Lastly, we have shown that transcriptional changes following exposure to e-cigarette vapour or in e-cigarette smokers show a similar trend to those in CS-exposed cells in the current study; this is in line with previous studies showing that that e-cigarette may induce both shared and distinct patterns of gene expression when compared to tobacco smoking [28, 45]. However, interestingly, and consistent with the pattern after CS exposure, the DEGs higher in expression at 24 h after e-cigarette exposure reached the peak of their expression at 4 h and goes down at 24 h, although still significant compared to Air. This suggests that, like CS, also e-cigarettes may elicit its transcriptomic effects at the early stages of exposure. While these similarities are interesting, further studies are needed, especially since the e-cigarette study was based on the use of a single donor, whereas the observational study in e-cigarette users was not a controlled study.

This study also has a number of limitations that need to be taken into account. Firstly, since we have not performed single cell RNA-seq the cell-specific mechanisms in response to cigarette smoke exposure are not directly explored. However, findings from the cellular deconvolution analysis showed that there might be a response initiated related to goblet cell hyperplasia after cigarette smoke exposure, which is line with existing literature [46, 47]. It will be interesting to establish a single-cell RNA-sequencing follow-up study that elucidates a detailed cell-type specific differences in altered gene expression upon cigarette smoke exposure. Furthermore, this study is performed with cell cultures derived from 8 different donors that were not matched in smoke history and with only one female included. We were therefore unable to investigate not only a possible sex effect in response to CS exposure, but also not able to compare responses of donors that were non-smokers, ex-smokers and current smokers. Such studies would have required inclusion of additional, adequately sized subgroups. Additionally, we found that many of the pathways identified in the current study had a number of overlapping genes indicating a common core list of genes that are likely regulating the majority of smoking related pathways.

## Conclusion

In conclusion, our study has provided novel insights in the epithelial transcriptional responses to CS immediately after exposure, and provides validation for the relevance of the findings in humans. These are important steps that are needed for the stepwise development of more complex models that will contribute to the reduction in the use of animal models.

## Supplementary Information

**Additional file 1.** Detailed method of the current study.

**Additional file 2.** List of differentially expressed genes and associated pathways after cigarette smoke exposure compared to Air.

## Data Availability

The datasets analysed in the current study are available from the corresponding author.

## Abbreviations

AP-1: Activator protein-1
ALI: Air–liquid interface
AhR: Aryl hydrocarbon receptor
ChIP-seq: Chromatin immunoprecipitation sequencing
COPD: Chronic obstructive pulmonary disease
CS: Cigarette smoke
DEG: Differentially expressed gene
FDR: False discovery rate
FC: Fold change
GSVA: Gene set variation analysis
IEG: Immediate early gene
CPM: Counts per million
NNLS: Non-negative least squares
Nrf2: Nuclear factor erythroid 2-related factor 2
PAH: Polycyclic aromatic hydrocarbons
PBEC: Primary bronchial epithelial cells
ROS: Reactive oxygen species
SD: Standard deviation
SVR: Support vector regression
UMAP: Uniform manifold approximation and projection
