# Supplementary material for: Early transcriptional responses of bronchial epithelial cells to whole cigarette smoke mirror those of in-vivo exposed human bronchial mucosa
Source: Respir Res. 2022 Sep 2;23:227. doi: 10.1186/s12931-022-02150-2 (PMC9440516; doi:10.1186/s12931-022-02150-2)

## METHODS

### Study population and sample selection

Primary bronchial epithelial cells (PBEC) were isolated from tumour-free resected lung tissue (n=8), expanded and stored in liquid nitrogen at the Leiden University Medical Center, Leiden, the Netherlands, as previously described.(1, 2)

### Primary bronchial epithelial cell culture

Cryopreserved PBEC were thawed at passage one in a T75 flask coated with 10 μg/ml BSA (ThermoFisher Scientific), 30 μg/ml Purecol (Advanced BioMatrix, San Diego, CA), and 10 μg/ml fibronectin (Promocell, PromoKine, Bio-connect, Huissen, The Netherlands), in serum-free keratinocyte medium (KSFM, Thermo Fisher Scientific) supplemented 100 U/mL penicillin (Sciencell Research Laboratories, Sanbio, Uden, The Netherlands), 100 μg/ml streptomycin (Sciencell Research Laboratories, Sanbio) and 2.5 ng/ml epidermal growth factor (EGF, ThermoFisher Scientific), 25 μg/ml bovine pituitary extract (BPE, ThermoFisher Scientic) and 1 μM isoproterenol (Sigma-Aldrich). After reaching ~90% confluency, cells were trypsinized in 0.03% (w/v) trypsin (ThermoFisher Scientific), 0.01% (w/v) EDTA (BDH, Poole, UK), 0.1% glucose (BDH) in PBS and seeded on permeable 12 mm PET transwell inserts with 0.4 μm pore size (Corning Costar, Cambridge, USA) and cultured as described in B/D complete (BEpiCM-b:DMEM (B/D)-medium (1:1; ScienCell Research Laboratories, Sanbio and STEMCELL Technologies, Köln, Germany, respectively), supplemented with Bronchial Epithelial Cell Growth Supplement (ScienCell Research Laboratories, Sanbio), 1 nM EC-23 (Tocris, Bio-techne Ltd, Abington, UK), 25 mM HEPES (Cayman Chemical, Hamburg, Germany), 100 U/mL penicillin and 100 µg/mL streptomycin (ScienCell Research Laboratories, Sanbio).(3) After reaching full confluence, apical medium was removed and cells were cultured at the ALI in the same medium as above with the EC-23 concentration increased to 50 nM. Media were changed three times a week and simultaneously the apical side of the cell layer was washed with warm PBS to remove excess mucus.

### Differentiation in air-liquid interface (ALI) and exposure to whole cigarette smoke/air

After 19-21 days of differentiation at the ALI, cell culture media was changed to B/D complete without hydrocortisone, and 48 h later cells were washed on the apical side with warm PBS in the morning after which they were exposed once to whole cigarette smoke (CS) or to air (Air) as a control. In brief, cells were exposed in modified hypoxic chambers for 4-5 minutes to freshly generated whole cigarette smoke (CS) from one cigarette using 3R4F reference cigarettes (University of Kentucky, Lexington, KY) or room air as a control after which smoke was removed by ventilation with incubator air during 10 minutes and cells were subsequently placed back in another incubator (detailed description of the cigarette smoke and air exposure set-up is provided in Amatngalim *et al*.(2)). Exposure to CS was performed in two independent experiments of 4 donors each on different days. This resulted in an exposure of four donors to CS equivalent to 2.1 mg cigarette smoke particulate (as assessed by particulate collected in filters) and the other four donors to 3.9 mg. Additionally, incubator controls were kept untouched in the cell culture incubator during the whole procedure to control for the total process of exposure. Directly after CS/Air exposure, the basal media of all transwells was replaced with pre-warmed B/D complete without hydrocortisone (T=0h). At 1h, 4h, and 24h hereafter basal media were collected and stored at -20ºC pending ELISA analysis, and cells were lysed by addition of 200 µl of lysis buffer to the transwell (Promega Benelux B.V. Leiden, the Netherlands) and the lysate was stored at -20ºC until RNA isolation.

### RNA extraction, sample preparation and sequencing

The total RNA was robotically extracted using the Maxwell tissue RNA extraction kit (Promega), quantified using the Nanodrop ND-1000 UV-Vis Spectrophotometer (Nanodrop Technologies, Wilmington, DE, USA) and stored at -80ºC until analysis by GenomeScan (Leiden, the Netherlands). RNA sequencing was performed with the cDNA fragment libraries by the Illumina NovaSeq6000 sequencer using 150 bp pair-end sequencing settings.

### Differential gene expression (DGE)

The GRCh38 (hg38) genome assembly was used to map the genome. Differential gene expression (DGE) was analysed using the edgeR package (version 3.30.3) in R (version 4.0.2). A linear model was used to explore the changes in gene expression upon cigarette smoke exposure. The response to cigarette smoke exposure of airway epithelial cells from healthy donors was compared with air exposure. To correct for multiple testing, the false discovery rate (FDR) was set to 5% using the Benjamini-Hochberg procedure. Logarithmic fold change (logFC) above 1 and below -1 was considered significantly upregulated and downregulated, respectively.

### Gene set variation analysis (GSVA)

Gene set variation analysis (GSVA) was performed on the differentially expressed genes upon cigarette smoke exposure using the GSVA package (version 1.36.2) in R (version 4.0.2).

### Pathway analysis

Pathway analysis was performed in an unbiased fashion using gprofiler (https://biit.cs.ut.ee/gprofiler/gost). The significantly up- and downregulated genes (FDR<0.05, FC >|2|) at 1h, 4h, and 24h were provided, separately, as ‘ordered query’ in gprofiler. ‘Homo sapiens (Human)’ was selected as the organism and the databases included in the gprofiler platform were Gene Ontology, KEGG, Reactome, WikiPathways, TRANSFAC, and CORUM. The top pathways associated with the differentially expressed genes were further analysed.

To investigate the gene expression pattern of the pathway associated genes, GSVA was performed. The Immediate-Early Genes (IEG)s (*FOS, FOSB, JUN, NR4A1, NR4A2, MCL1, ATF3, EGR1*) were identified from the existing literature as the list is not updated in the renowned databases such as KEGG or GeneOntology. The genes associated with AP-1, ferroptosis, and AhR were collected from the GeneOntology (GO:0035976), KEGG (KEGG:04216), and WikiPathways (WP2873) databases, respectively. Gene set variation analysis was performed for each pathway using the associated genes identified from the databases, regardless of the outcome of the differential gene expression analysis. GSVA package (version 1.36.2) in R (version 4.0.2) was used to perform this analysis.

The nuclear factor erythroid 2-related factor 2 (Nrf2) pathway was explored using the publicly available RNA sequencing dataset of Nrf2-siRNA (small interfering RNA) treated A549 cells (GSE113519). The siRNA-Nrf2 dataset was analysed using the edgeR package (version 3.30.3) in R (version 4.0.2). Differentially expressed genes (FDR<0.05) with logFC below -1 were considered downregulated and associated with Nrf2. Furthermore, positions of the Nrf2-associated genes were matched (partially or completely) with Nrf2-binding sites in close-proximity (±50,000 bp) based on previous ChIP-seq analysis (GSE75812). To estimate the variation of pathway activity, signature expression of these Nrf2-associated genes in the current study was analysed using GSVA package (version 1.36.2) in R (version 4.0.2).

### CXCL8 ELISA

The protein encoded by *CXCL8*, a pro-inflammatory protein also known as interleukin-8 (IL-8), was measured with the use of the Recombinant Human IL-8/CXCL8 Protein ELISA kit of R&D (Abingdon, UK) and performed according to manufacturer’s instructions and analysed with the use of 4-parameter logistic curve fitting in Prism 8 (version 8.1.1).

### Cellular deconvolution

To determine the differences in cell-type composition, gene expression signatures for basal, ciliated, and secretory (club and goblet cells) were used from previously published mRNA expression levels obtained by single cell RNA sequencing.(4) Genes were selected using data from bronchial biopsies which best represented the unique profiles of each cell types. Next, bulk deconvolution was conducted using both support vector regression (SVR) and non-negative least squares (NNLS) method.(5) Cell-types which were abundant less than 5% were excluded and GSVA was performed on basal, ciliated and club/goblet cell-types.

**FIGURE LEGENDS**

**Figure S1: Gene expression at 1, 4 and 24h after cigarette smoke exposure in ALI-PBEC cultures using two doses of cigarette smoke.** ALI-PBEC cultures derived from 8 different donors were exposed to cigarette smoke in two independent exposures. Cultures from 4 donors were exposed to 2.1 mg (CS^low^) of cigarette smoke exposure and the other 4 donors were exposed to 3.9 mg (CS^high^). Differential gene expression was performed between CS^high^ and CS^low^ (adj. p-value<0.05, FC>|2|). The differentially expressed genes between CS^high^ and CS^low^ at (A) 1h, (B) 4h and (C) 24h are represented in the volcano plot. (D) The heatmap represents the DEGs of 1h and 4h after CS exposure.

**Figure S2. Expression of immediate-early genes (IEGs) at 1h, 4h and 24h after whole cigarette smoke exposure in primary bronchial epithelial cells differentiated at air-liquid interface.**

The time series line graph depicts the expression of the identified eight IEGs (*FOS, FOSB, JUN, NR4A1, NR4A2, MCL1, ATF3, EGR1*) from the literature. The points are the mean expression of the respective genes (n=8) and the error bar indicates standard deviation. Genes which were significantly different after whole cigarette smoke exposure compared to Air (p<0.05) are shown by * symbol. *=p<0.05, **=p<0.01, ***=p<0.001.

**Figure S3: GSVA of genes associated with Nrf2 pathway which are in proximity to the binding sites of antioxidant responsive elements.**

Nrf2 is a transcription factor that regulates gene expression by binding to antioxidant responsive elements. GSVA of Nrf2 associated genes which are close in proximity to these binding sites (±50,000 bp) are shown here. The error bar indicates the standard deviation (n=8). Gene sets which were significantly different after cigarette smoke exposure compared to Air (p<0.05) are shown by the * symbol. ***=p<0.001.

**Figure S4. Cell-type deconvolution analysis of ALI-PBEC cultures exposed to whole cigarette smoke or air.**

Cell-type deconvolution was performed using two different methods, support vector regression (SVR) and non-negative least squares (NNLS). Proportions of different cell types are depicted with the bar-plot. The vertical error bar indicates standard deviation.

**REFERENCES**

1. van der Does AM, Heijink M, Mayboroda OA, Persson LJ, Aanerud M, Bakke P, Eagan TM, Hiemstra PS, Giera M. Dynamic differences in dietary polyunsaturated fatty acid metabolism in sputum of COPD patients and controls. *Biochim Biophys Acta Mol Cell Biol Lipids* 2019; 1864: 224-233.

2. Amatngalim GD, Schrumpf JA, Dishchekenian F, Mertens TCJ, Ninaber DK, van der Linden AC, Pilette C, Taube C, Hiemstra PS, van der Does AM. Aberrant epithelial differentiation by cigarette smoke dysregulates respiratory host defence. *Eur Respir J* 2018; 51.

3. Schrumpf JA, Ninaber DK, van der Does AM, Hiemstra PS. TGF-β1 Impairs Vitamin D-Induced and Constitutive Airway Epithelial Host Defense Mechanisms. *J Innate Immun* 2020; 12: 74-89.

4. Braga FAV, Kar G, Berg M, Carpaij OA, Polanski K, Simon LM, Brouwer S, Gomes T, Hesse L, Jiang J. A cellular census of human lungs identifies novel cell states in health and in asthma. *Nature medicine* 2019; 25: 1153-1163.

5. Avila Cobos F, Vandesompele J, Mestdagh P, De Preter K. Computational deconvolution of transcriptomics data from mixed cell populations. *Bioinformatics* 2018; 34: 1969-1979.


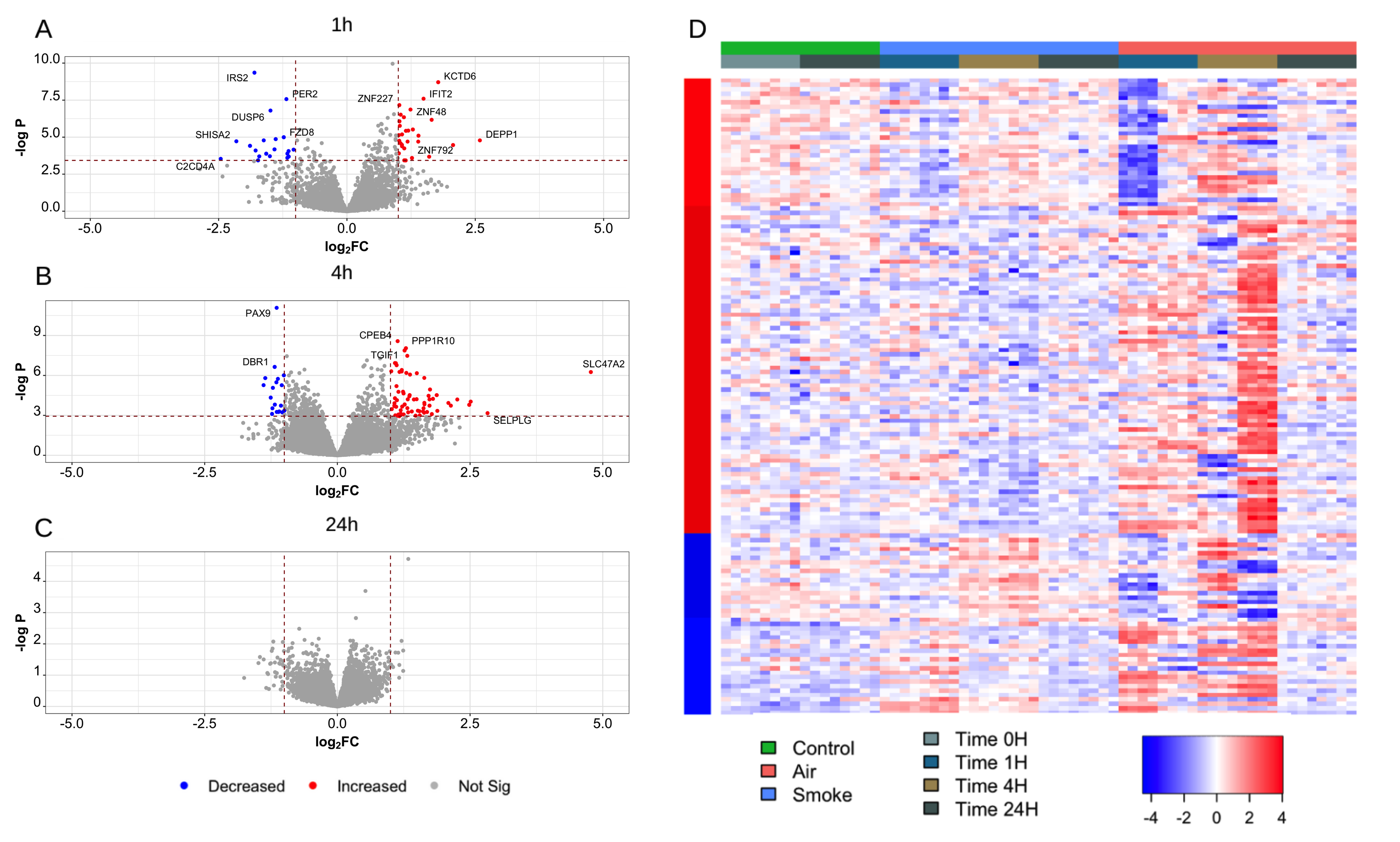

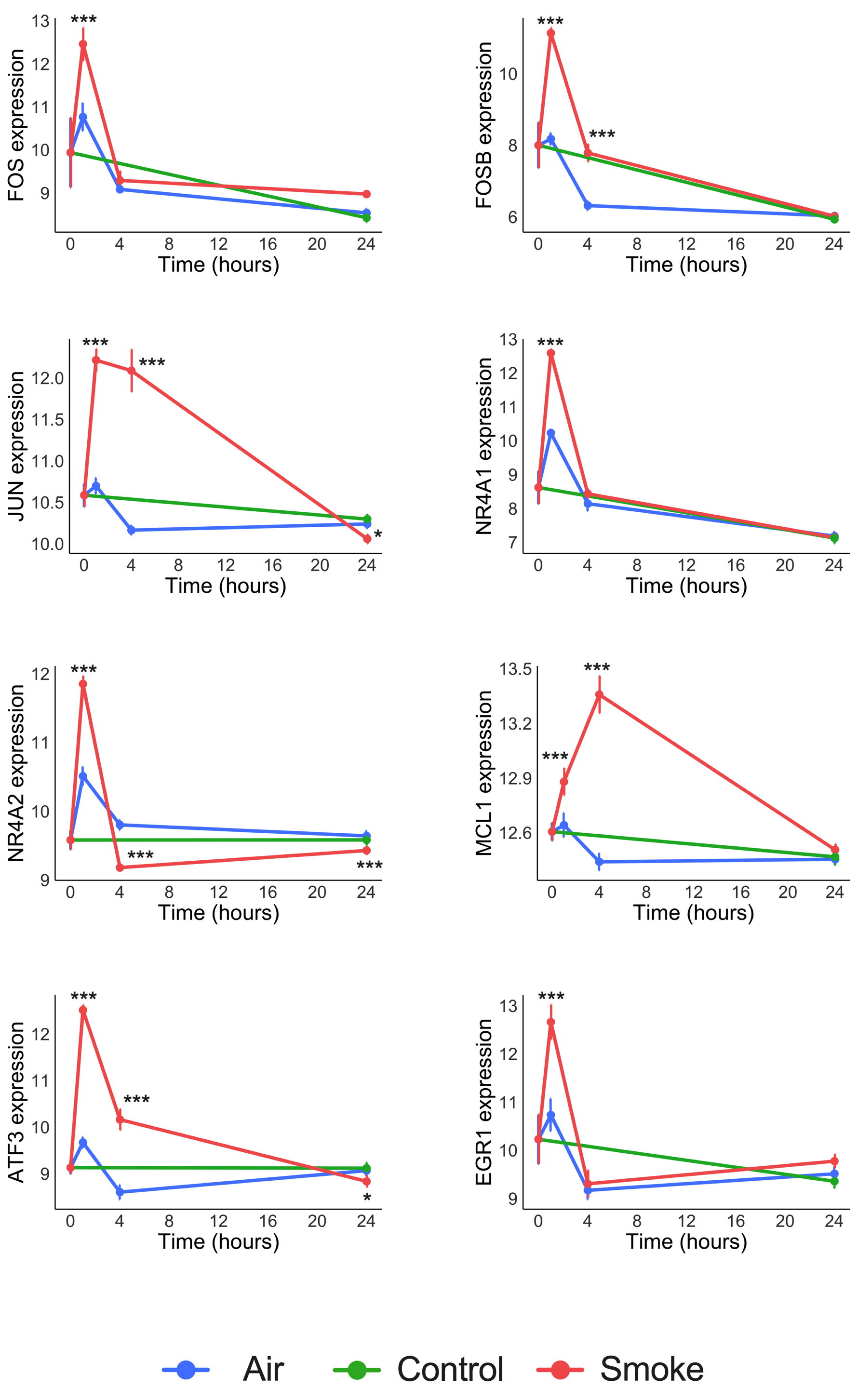

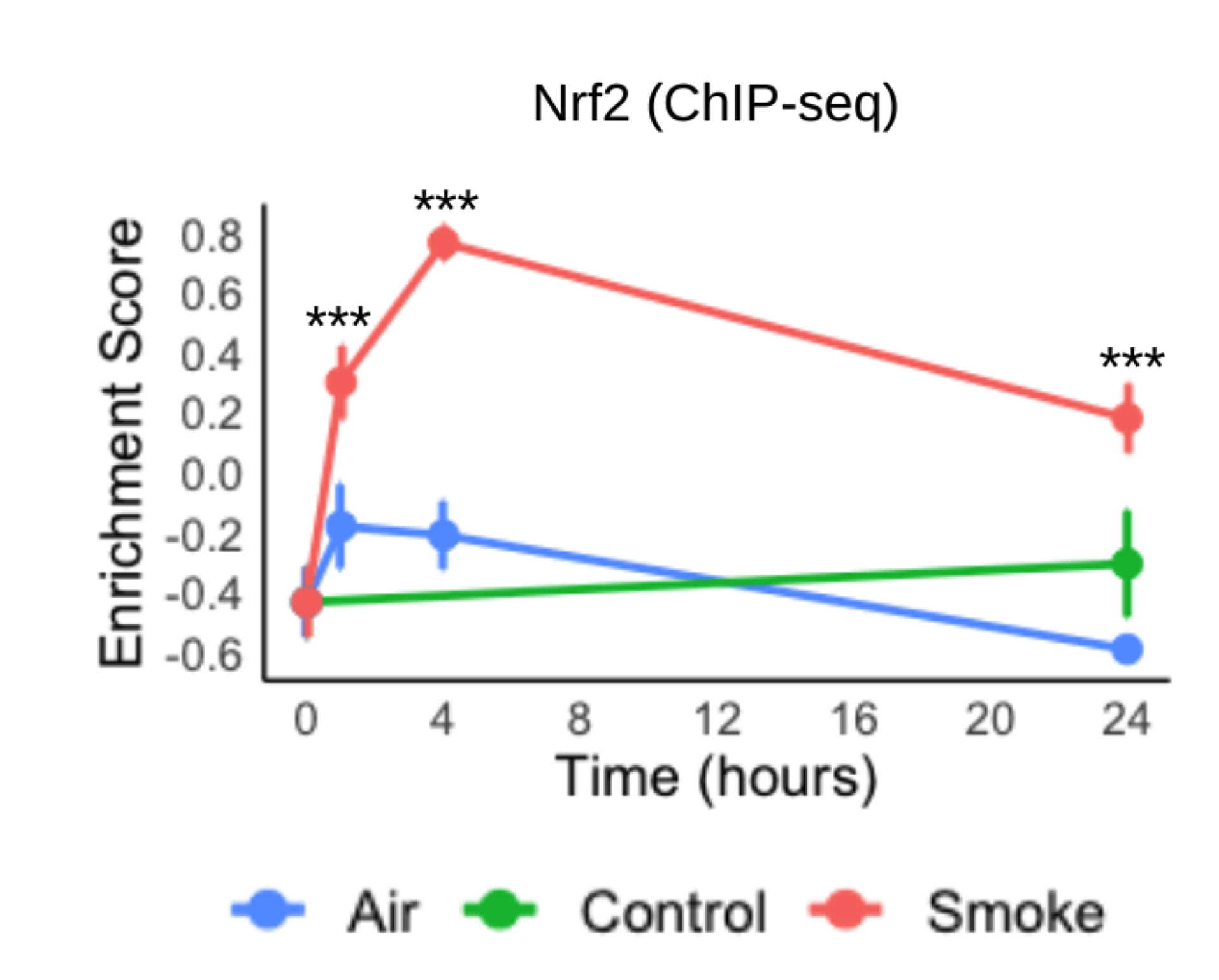

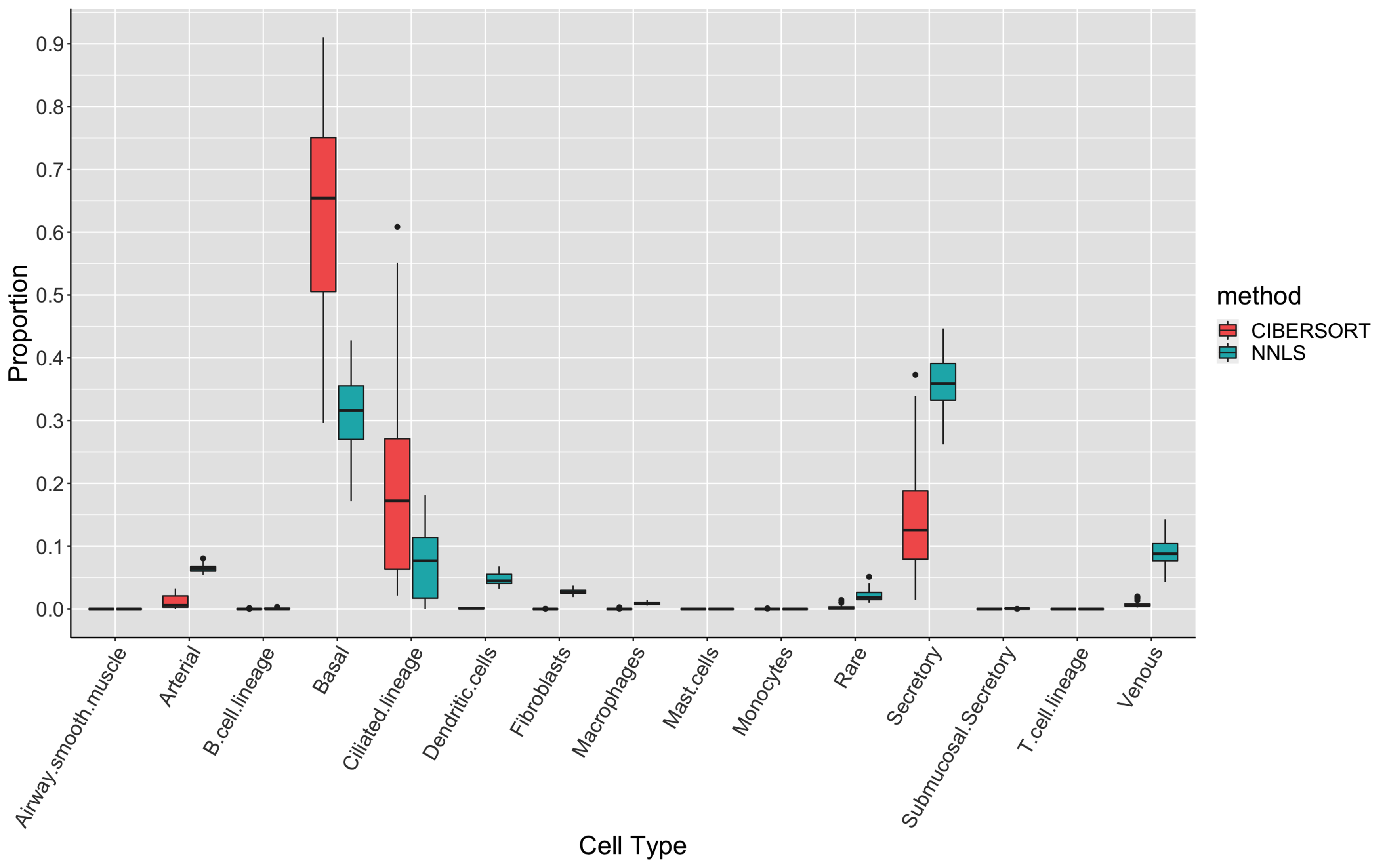

Supplement: Supplementary file 1 — Additional file 1. Detailed method of the current study. [file 12931_2022_2150_MOESM1_ESM.docx]
